# Dermatology

**Published:** 1876-02

**Authors:** 


### VII. Dermatology.

1. *Proofs of the Dartrous Diathesis furnished by Comparative Pathology.* Mégnin. (*La France Médicale*, Dec. 4, 1875.)

The reporter, a veterinary surgeon of the French military service, assigns the cutaneous disorders of the lower animals to three grand divisions: accidental, parasitic, and constitutional. He finds that the degree of domesticity has a modifying influence, those longest under subjection, as the dog, presenting cutaneous affections more frequently than the horse and the ox, which are less domesticated.

Lafosse observed psoriasis in the dog during four generations, the disease being transmitted from parent to offspring. Mégnin gives the details of the following case:

A young ox, of fine appearance and in good flesh, had an eruption of miliary vesicles, upon three years of age, in the spring of 1872. The eruption became rapidly confluent, and was seated exclusively upon the lumbar region, though it descended irregularly from the vertebral line on each side, to the extent of four or five fingers in breadth. There was moderate itching, followed in succession by the formation of granular crusts among the hairs, and a very abundant epidermic exfoliation, accompanied by alopecia. At the onset of winter, the animal presented in the centre of the lumbar regions, a denuded, smooth, rose-colored and shining surface, covered with large laminated, friable scales, readily detached by friction, and unaccompanied by itching or exaggerated sensibility. Sulphur and other preparations seemed to limit the extension of the disease, but did not produce a new growth of hair.

In the spring of 1873, there was recrudescence of the disease, the patch becoming larger in every direction, the newly invaded surfaces successively exhibiting vesicles, granular crusts, desiccation of the sero-albuminous secretion of the vesicles, loss of hair, and extensive epidermic exfoliation. Treatment had no greater effect than formerly. In the spring of 1874, there was a third attack, the disease affecting a surface extending from the throat in front to the base of the tail behind, and descending laterally to the middle of the costal region. The affection pursued the same course as formerly, but curiously enough, it respected all the parts colored by pigment and covered by red hairs. In July, the alopecia and exfoliation left the superior half of the trunk almost entirely bare.

When examined at this time, almost all of the superior part of the body presented an irregular surface, deprived of hair, and covered with large diaphanous laminated scales, adherent by one or two borders (third stage of eczema). In front, the affection was still in its initial period, the hairs were still in situ, but bristling and surrounded by granular crusts. At the base of the hairs could be seen the reddened skin, superficially ulcerated, and presenting all the signs of the second stage of eczema. In the midst of this dry, eczematous and laminated surface, were islets of sound skin, covered with red hairs. In all the affected parts, the skin preserved its normal suppleness and thickness, hence M. considered it to be an eczema, of what the French call the " herpetic " class.

Microscopic examination demonstrated that the superficial parts of the derma were alone affected, and the absence of parasites.

What follows is interesting, in view of the fact that the English and American schools of dermatologists are disposed to doubt the existence of the dartrous diathesis, upon which depends the pathology of the French view. According to the latter, the symptoms detailed above, point to a constitutional diathetic affection.

Veterinary practice has this advantage over the treatment of diseases in the human body: the affected subjects may be killed at any stage of a malady to rectify a diagnosis, or to note the extent of the disease. In the instance of the ox whose case is described above, no disorder of the splanchnic viscera, nor of the vascular and muscular systems, was established post-mortem. *All* the bones, however, on section, presented a striking discoloration, which was noticed even by the butcher. Brown concentric rings also appeared, equal in number to the recrudescences of the malady; and suggested the well-known experiments of Flourens on chicken bones. Microscopically, the coloration of these zones appeared to be exclusively due to deposition of pigment granules quite analogous to those found in the skin. This fact tended to show a profound alteration of nutrition, effected by a disease whose principal manifestation was in the skin, *i. e.*, a constitutional disease.

The paper concludes as follows: In the view of Hardy, chronicity, tendency to relapse, simultaneous existence or alteration of lesions of skin and mucous membranes, and heredity (occasionally established), point to a morbid principle affecting the whole economy, and generating the dartrous diseases. The facts, cited above, give additional proof of the impregnation of the entire economy with [illegible]ne morbid principle, which is called the dartrous vice. It also adds to the experiments of Lafosse, referred to above, as showing the inanity of the anatomical theory, which can only discern in the dartrous diseases a local inflammation. This theory is still held by Devergie and some pupils of Cazenove.

It must be admitted that the advocates of a dartrous diathesis possess, in the details collected by Mégnin, the strongest argument yet advanced in support of their views.

2. *Pellagra.* BÉHIER. (*Le Progrès Médical*, Nov. 6.)

Two cases of sporadic pellagra having been discovered in the Hotel Dieu, Professor Béhier thus describes the cutaneous lesions:

These vary in appearance according to the period of observation. The cutaneous disorder has been properly called pellagrous erythema, for at the outset it is decidedly erythematous and, later, when there is profound alteration of the skin and the disease has become inveterate, the cutaneous manifestations are varied. The integument becomes dry, fissured and furrowed; later, a species of ichthyosis is developed (Strombio). After several successive attacks, warty and even horny tumors (Coldesini) appear. It is the Italian authors who have especially dwelt upon these exceptional forms, and several among them are more remarkable for their fancifulness than for their exactness of description. The eruption is chiefly located upon the hands and feet, but is also to be found upon the face and the lateral portions of the neck—situations, in short, which are [illegible]t protected by clothing. When stockings are worn, the erup[illegible]on will [illegible]pear where any holes are to be found; and in Landes the parts of the leg protected by the hunting leggings remain unaffected (Bouchard).

3. *Generalized Melanosis.* BERGERON. (*La France Méd.*, Nov. 13, '75.)

B. reports the case of a young silversmith, 21 years old, pale and emaciated, having reddish patches diffused over the skin—principally of the face—and slightly discolored mucous surfaces. He had never suffered from scrofula, syphilis, or other grave disease, and his hereditary antecedents were excellent. A series of painful tumors developed upon the surface of the integument of his chest, some attaining the size of the fist. In the first, the integument which covered it, was unaltered in color, but soon ulcerated, and gave exit to a bluish-black, semi-solid matter, exactly resembling wet powder. Spontaneous closure of the orifice occurred, and was succeeded by reformation of the tumor. This second became as large as two fists, and, after a drainage tube had been inserted with no results, it was opened and again gave exit to blackish contents. Six similar tumors appeared in rapid succession soon after, but these were unaccompanied by pain. The first, having increased considerably in size and become more painful, was removed by the knife, no ganglionic enlargement having been found in the neighborhood.

On the 27th of October, spontaneous disappearance of two of these tumors had occurred, without the intervention of physician or surgeon.

These tumors were evidently situated in the subcutaneous cellular tissue, having but slight connection with the subjacent fasciæ. They were indolent, indurated, irregular and rough. The skin glided freely over their surface, but through it could easily be seen the black color characteristic of melanotic tumors. No lesion or tumor of the internal organs could be discovered. The only constitutional symptoms evident were: emaciation, feebleness and anorexia.

Though melanosis and melanotic cancer have certain symptoms in common, one need never be mistaken for the other, since the distinction between them has been clearly defined by Nélaton. The case reported is interesting, as no record has yet been made of the spontaneous disappearance of similar growths, nor of their occurrence in so young a subject.

4. *A Case of Argyria.* B. RIEMER. (*Arch. d. Heilkunde*, 1875, No. 4.)

A man, 43 years old, was treated for tabes with nitrate of silver, and had taken during two years about 550 grains. The whole integument, especially of the face, had assumed a gray hue, when he died of pulmonary disease. Silver was found in almost all the internal organs, but most abundantly in the skin. Numerous grains of pure silver were deposited in the connective tunics of the corium, chiefly in the vicinity of the small vessels, and in the tissues of the sweat-glands. No silver was found in the layers of the epidermis and the tunics of the lymphatic vessels. The writer is of the opinion that the deposition of silver is purely mechanical. By the secretory organs of the integuments, the reduced metal is removed from the blood, and, since it cannot pass through the epidermis, it remains beneath, and there accumulates.

5. *Feigned "Erythema Gangrænosum."* Fox. (*Lancet*, Jan. 16.)

The author states that he has always regarded the disease which answers to the term given above, with grave suspicion. In almost all cases it is feigned.

A stout and healthy-looking servant girl exhibited, in various parts of her body, long red patches; with scabs forming on the outer edge of each patch, leaving a narrow, red rim around it. Some of those broke up into irregular patches of dead white skin, surrounded by a reddish hole. Over the parts which did not become red, a slight, thin, brownish-yellow crust formed. Anæsthesia seemed to exist where the dead-white areas of skin were seen, with elevation of temperature. On the lower extremities, the general appearance of the eruption gave the idea that some corrosive liquid had run down the front of the leg, and caused the skin to lose its vitality.

When under observation in hospital, the dead-white patches began to assume a yellowish tinge, some becoming covered with a dark honey-colored crust, and some exhibiting a loosening of the cuticle. Deep, sharply-cut ulcers, surrounded by slightly red inflamed margins, were formed on the surface of one leg. When the crusts mentioned above fell, a red blush was left upon the integument. Patches of white cuticle on the breast when rubbed off, left a weeping surface of true skin, which soon scabbed over. The ulcers healed readily.

This was decided to be a case of malingering on the following grounds: 1. The general health was undisturbed. True erythema gangrænosum occurs in the moribund condition, with profound alteration of nutrition. 2. The onset and course of the disease were those of severe blistering, not of a gangrenous disease. There was no livid sphacelated surface ; a healthy reparative process was speedily set up. 3. The distribution of the patches was peculiar. They were located, with one exception, on the left side, in parts most accessible to the right hand. (The author does not observe that the patient was right-handed.) 4. The statements of the patient relative to anæsthesia conclusively showed deception. 5. The effect of pseudo-physic in the case confirmed the view taken above. (The patient had improved on a solution of sugar and water taken internally, the wounds being dressed with the simplest unguent.)

No reasons for practicing the deception were discovered, nor was the irritating agent known. The author supposed hydrochloric acid to have been used.

6. *Use of the Induced Currents in Zona.* Dr. Faugue. (*Tribune Médicale*, 1875, page 364.)

The author, (in his inaugural thesis, Paris, 1875, No. 37), recommends, according to the observations and practice of Dr. Picot (of Tours), the use of the induced currents for the pains, often so persistent, of zona. The positive pole should be placed at the side of the spine, and the negative pole upon the painful points.

7. *Treatment of Acne by Soft Soap.* HEBRA—LALLIER. (*La France Médicale*, No. 20, 1875.)

On retiring at night, the patient takes a small quantity of the soft soap upon the end of the finger, and lightly rubs with it the parts affected with acne. This delicate layer of soft soap is allowed to remain in place during the night, and is removed with tepid water in the morning. A single application is not sufficient to produce the desired effect, and hence the friction should be repeated for four consecutive nights. On the fifth day, this application is suspended, and the vapor of steam is substituted for four days. Where this is intolerable or impracticable, an emollient wash of bran or starch water is employed.

[We have found the bran water most efficacious in the pustular forms of the disease. If the face is affected to such an extent that coughing or sneezing produces an intense temporary congestion of the parts, the vapor of steam seems occasionally to favor this occurrence.—ED.]

Guillot concludes from his experience that the treatment of the different forms of acne by friction with soft soap, followed by the topical application of powders, douches or emollient lotions, is easily conducted, does not in general bring about inflammatory complications, and, in confluent cases, will relieve in from six weeks to two months. It is most serviceable in tubercular, pustular or punctate acne, whether these forms be isolated or concurrent; and although the roseolous variety may seem to be aggravated by the treatment, it is ameliorated, particularly when accompanied by a few simple or tuberculated papules. In moist sebaceous acne it is less available. There is no record of its employment in varioliform acne.

The treatment is highly satisfactory in its results, but patients must be cautioned to use only a small quantity of the soft soap at first, as an excess is sure to excite inflammatory action.

8. *Treatment of Infantile Eczema.* CASPARI. (*Bulletin de Thérapeutique*, page 29, 1875.)

Lime water is recommended in infantile eczema capitis and impetigo faciei. The dose varies with the age of the child, from 150 to 300 grammes. With older children, where the confluence of the disease excites pain and restlessness, the lime water is given internally, mixed with pure or diluted milk. The remedy is particularly praised in chronic and rebellious forms, which are ordinarily ameliorated after eight days of medication. External treatment is resorted to only when the secretion is exceedingly irritating, and then Caspari dusts the parts well with carbonate of magnesia. He recommends parents who are poor to wash the diseased surfaces once or twice daily, with a weak decoction of wood ashes.

9. *Silicate of Potassa in Erysipelas.* ALVARENZA. (*Centralblatt f. d. Chir.*, No. 35, 1875.)

A solution containing one part of the silicate to one or ten of water, is used externally, the silicate containing one part of silicic acid to four parts of the potassic solution. The cure seemed to be rapid in proportion to the concentration of the solution. The author has collected notes of forty-eight clinical cases thus treated, and many others in private practice, having, on seven different occasions, relieved the disease in three days, using a preparation containing one-third or one-fourth of soluble glass.

10. *Collodion for Freckles.* (*La France Médicale*, Nov. 20.)

The mercurial preparations ordinarily employed for the removal of freckles, contain either the bichloride or the cyanide—sometimes both—and are not without danger. Good results may be obtained without resorting to these, by mixing forty-five parts of collodion with five of pure alcohol and one of the essence of lemon. Finally, one part of the sulphophenate of zinc is reduced to a very fine powder and mchanically mixed with the solution.

11. *External Application of Carbolic Acid in Cutaneous Diseases.* BERNDGEN. (*Allg. Med. Central Zeitung*, Nov. 20, 1875; *Archives of Derm.*, January.)

In chronic eczema, B. employs acid. carb. cryst., 5.0; spts. vin. dil., 10.0; aq. destill., 120.0—applied with a brush every morning. In recent cases, 20 per cent. watery or oily solution is used, either as a wash or dressing. It is considered too irritating for the acute stage. He claims great benefit from this topical medication in psoriasis; and, in inveterate cases, recommends acid. carbol., 2.5; spts. vin. dil., aq. dest., *aa* 10.0. It should be discontinued in from three to four days. In prurigo, five per cent. solutions are advised.

12. *Treatment of Alopecia Areata.* J. H. STOWERS. (*British Med. Jour.* August, 1875.)

The author has repeatedly used, with good success, the liquor ammon. caust. and spir. terebinthinæ. The first named remedy is to be rubbed into the scalp with a piece of flannel twice daily, or less often if it causes much irritation. If the ammonia is not tolerated well, he advises the use of turpentine. This treatment must be continued for several months.

